# RNA Deep Sequencing Reveals Differential MicroRNA Expression during Development of Sea Urchin and Sea Star

**DOI:** 10.1371/journal.pone.0029217

**Published:** 2011-12-28

**Authors:** Sabah Kadri, Veronica F. Hinman, Panayiotis V. Benos

**Affiliations:** 1 Lane Center for Computational Biology, Carnegie Mellon University, Pittsburgh, Pennsylvania, United States of America; 2 Department of Biological Sciences, Carnegie Mellon University, Pittsburgh, Pennsylvania, United States of America; 3 Department of Computational and Systems Biology, University of Pittsburgh, Pittsburgh, Pennsylvania, United States of America; Inserm U869, France

## Abstract

microRNAs (miRNAs) are small (20–23 nt), non-coding single stranded RNA molecules that act as post-transcriptional regulators of mRNA gene expression. They have been implicated in regulation of developmental processes in diverse organisms. The echinoderms, *Strongylocentrotus purpuratus* (sea urchin) and *Patiria miniata* (sea star) are excellent model organisms for studying development with well-characterized transcriptional networks. However, to date, nothing is known about the role of miRNAs during development in these organisms, except that the genes that are involved in the miRNA biogenesis pathway are expressed during their developmental stages. In this paper, we used Illumina Genome Analyzer (Illumina, Inc.) to sequence small RNA libraries in mixed stage population of embryos from one to three days after fertilization of sea urchin and sea star (total of 22,670,000 reads). Analysis of these data revealed the miRNA populations in these two species. We found that 47 and 38 known miRNAs are expressed in sea urchin and sea star, respectively, during early development (32 in common). We also found 13 potentially novel miRNAs in the sea urchin embryonic library. miRNA expression is generally conserved between the two species during development, but 7 miRNAs are highly expressed in only one species. We expect that our two datasets will be a valuable resource for everyone working in the field of developmental biology and the regulatory networks that affect it. The computational pipeline to analyze Illumina reads is available at http://www.benoslab.pitt.edu/services.html.

## Introduction

The developmental program, the process that creates a multicellular organism from a single cell, involves gene regulation at various levels – transcriptional and post-transcriptional. microRNAs (miRNAs) are one such class of small (∼22 nts), non-coding RNA molecules that regulate protein coding gene expression post-transcriptionally. miRNAs typically target 3′ UTRs of protein coding genes, and usually downregulate their expression by affecting their protein levels [1], either by inhibiting mRNA translation, or by increasing its degradation rate [2], [3]. miRNA genes are generally transcribed by RNA polymerase II, by their own promoters [4], [5], or as parts of introns of protein coding genes [6], [7], [8]. The primary transcripts are processed into characteristic RNA stem-loop structures, which are further processed into ∼22 nt long duplexes in the cytoplasm by the RNAse III enzyme, *Dicer* [9], [10], [11]. The mature miRNAs typically have relatively higher steady-state levels than their corresponding miRNA*. However some miRNA* reach substantial levels and are known to have regulatory roles [12].

The first miRNAs, *lin-4* and *let-7* were discovered in *C. elegans*, as regulators of developmental timing [13], [14], and since then, miRNAs have been implicated in many developmental and tissue differentiation processes [15], [16]. miRNAs have been found in all animal lineages, although specific miRNAs have been lost and gained during evolution [17], [18]. Some orthologous miRNAs are associated with conserved expression in similar tissues, which may suggest conservation of function [19].

The sea urchin, *Strongylocentrotus purpuratus* and the sea star, *Patiria miniata* are used as model organisms for developmental and evolutionary studies, due to their phylogenetic position (invertebrate deuterostomes), and their well-characterized transcription factor gene networks. Despite the intense research that has been devoted to their developmental transcriptional pathways [20], [21], [22], [23], little is known about miRNA expression in these two organisms, especially during their early developmental stages. In early work, Pasquinelli *et al.* [24] examined the expression of the highly conserved *let-7* miRNA in 14 species from 8 phyla, and found that only sea urchin embryos lacked mature transcripts for the miRNA. More recently, Song *et al.* [25] showed that the main genes involved in the RNAi pathway are expressed in sea urchin embryos, and Wheeler *et al.* [26] found 45 miRNAs to be expressed in the adult sea urchin using 454 sequencing. They also sequenced a species of sea star, *H. sanguinolenta* and found 42 miRNAs in this sea star adult. miRBase (v. 17, April 2011) contains 64 entries for *S. purpuratus* miRNAs (including miRNA* species) [26], [27]. Since developmental transcription factor gene networks are very detailed in these organisms (more than in any other echinoderm species), a systematic overlay of miRNA level regulation will provide invaluable insight into the cumulative effects of transcriptional and post-transcriptional regulation on developmental wiring.

In this paper, we present for the first time, concrete evidence that many small non-coding RNA genes (including miRNAs) are expressed in high-numbers in the early developmental stages of two distantly related species, *S. purpuratus* and *P. miniata*, which last shared a common ancestor almost 500 million years ago (MYA) [28]. The goal of this study is to determine the pool of miRNAs involved in development of these two echinoderm species. We sequenced small RNA libraries of mixed population embryos from each of these echinoderms using Illumina Genome Analyzer (Illumina, Inc.), which provides a better depth of sequencing compared to 454. In the future, it will be extremely interesting to study stage-specific expression of these miRNAs. Comparison of the two sequenced datasets showed that a large number of miRNAs are expressed during development in the two species. Most of the identified miRNAs have homologs in other species, but a number of novel (echinoderm-specific) miRNAs were also identified. The data reported here will provide a valuable resource for evolutionary comparisons across a broader distance in the phylogenetic branch of deuterostomes, and this can help complete the puzzle of developmental gene regulatory networks in these two model organisms.

## Results and Discussion

### A rich population of non-coding RNAs is expressed in sea urchin and sea star embryos

High-throughput sequencing data (Illumina Genome Analyzer, Illumina, Inc.) corresponding to small RNAs were collected from a mixed embryonic population, individually from *S. purpuratus* (sea urchin) and *P. miniata* (sea star) as described in Methods. According to the Illumina protocol, the method specifically targets small RNAs with 3′ hydroxyl group, so the RNAs processed by *Dicer* and other RNA processing enzymes are preferentially sequenced with this method. A collection of publicly available programs and in-house made scripts were used to parse the Illumina reads, and quantify known and novel miRNA gene expression (see Methods).

Illumina sequencing of the small RNA libraries returned ∼13 million reads for sea urchin and ∼9.8 million reads for sea star embryos (**Table 1**). After removal of low quality 3′ ends and linker sequences, the remaining reads (∼11.6 and ∼9.01 million reads from sea urchin and sea star, respectively) were collapsed into “tags” based on sequence identity (see Methods). This process resulted in a total of ∼2.5 million tags from each species (**Table 1**).

**Table 1:** Summary statistics of sea urchin and sea star deep sequencing data, and annotations.

	*S. purpuratus*	*P. miniata*
Genome	800 Mb	500 Mb
Total number of reads	12,907,171	9,760,097
Reads mapped to genome	9,401,944	N/A
Tags (collapsed reads)”	2,486,028	2,513,198
Reads mapped to:		
tRNAs	7,550	33,551
rRNAs	288,036	319,035
snRNAs & snoRNAs	6,217	1,805
miRNAs (conserved)	376,007	48,320
miRNAs (potentially novel)	5,834	281
Number of conserved miRNAs	47	38
Potentially novel miRNAs (miRDeep)	11	3

Note that the number of reads for non-coding RNAs, such as tRNAs, rRNAs, snRNAs, snoRNAs and miRNAs, are for the length range 17–26 nts. For discovery of conserved miRNAs in the libraries, only tags with more than 2 reads were used, whereas, for potential novel predictions, tags with more than 5 reads were used.

**Figure 1:**
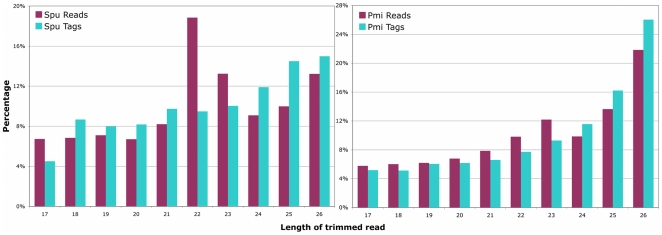
Length distributions of sea urchin and sea star reads. Histogram of length distribution of reads and tags in sea urchin and sea star small RNA Illumina libraries. The peak corresponding to the typical length of a miRNA is seen at 22 nts in sea urchin, but this peak is not as enhanced in the sea star library. *Spu: Strongylocentrotus purpuratus; Pmi: Patiria miniata*.

We focused on sequences of length 17–26 nts, since this is the typical size class expected for miRNAs. The histograms of the corresponding length distributions of reads and tags show similar trends between the two species (**Fig. 1**). In the sea urchin reads, there is a peak of relatively highly expressed sequences at 22–23 nts (corresponding to the typical length of a miRNA) (**Fig. 1**). The quality of the RNA was checked using a Bioanalyzer (**Figure S1**), before and after adapter ligation, and indicated that the RNA was preserved (For more details, check Methods).

**Figure 2:**
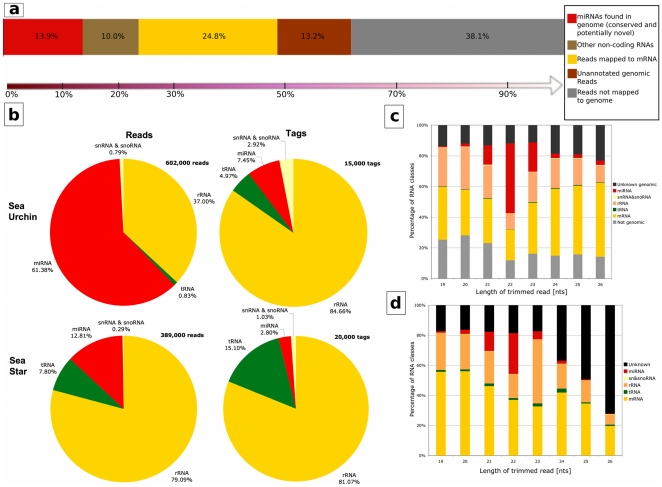
Distribution of annotated reads in small RNA libraries. (**a**) Bar showing the distribution of annotated reads 17 to 26 nts in length, for sea urchin. (**b**) Fractional distribution of non-coding RNAs in sea urchin and sea star embryonic small RNA libraries. Mapping of the annotated classes to reads and tags, shows the relative abundance (frequency) of each class per tag. All classes of non-coding RNAs compared were mapped to reads of lengths 17 to 26 nts. *Spu: Strongylocentrotus purpuratus; Pmi: Patiria miniata*.

Presently, *S. purpuratus* is the only echinoderm with a sequenced genome [29]. About 62% of the 17–26 nt sea urchin reads mapped to the genome (**Fig. 2a**) (81%, if reads of all lengths are considered). The reads that do not map to the genome could be the result of sequencing errors or genome quality. Since the sea star genome is unavailable, we assign all unmapped reads to the “unknown” category (**Fig. 2d**). Similarity searches against miRNAs and other known RNAs (coding and non-coding genes) were performed (see Methods). Approximately one quarter of the 17–26 nt long reads map to non-coding RNAs (14% to miRNAs and 10% other non-coding RNAs), another one quarter are mRNA degradation products, while 13% of reads map to the genome, but do not map to any annotated regions (**Fig. 2a**). **Fig. 2c & 2d** show the RNA composition of individual lengths in this size range in the sea urchin and sea star respectively. The 22 nt long sea urchin reads were most enriched for miRNAs, while this trend was not seen in the sea star library. All the size classes show an almost uniform distribution of mRNA and rRNA partial reads. The un-annotated reads could be attributed to the relatively poor annotation quality of the sea urchin genome, or to large-scale transcription as it has been observed in other species [30], [31], [32]. For example, a recent report showed that most intergenic reads are found near transcription start or termination sites [33].

The relative abundance of the reads and tags that map to various non-coding RNAs varies substantially between sea urchin and sea star (**Fig. 2b**). This is particularly true for miRNAs, where 61.4% of the sea urchin reads (17–26 nts) map to miRNA sequences compared to 12.6% of sea star reads. For sea urchin embryos, the miRNA reads collapse to ∼1,000 tags (that correspond to 42 miRNA genes), indicating a high expression of the miRNA genes (reads/gene average: 3,800; median: 413; 14 genes have >1,000 reads). By contrast, we found that a relatively higher number of sea star embryonic reads are mapped to (parts of) tRNA and rRNA genes (1.5% compared to 0.001%) (7.7% and 77.9% compared to 0.8% and 37% respectively) (**Fig. 2b**). This may reflect a sampling bias, or may indicate that fewer miRNAs are expressed in sea star embryos compared to sea urchin embryos. We found miRNA* species for most miRNAs, and in some cases, the miRNA* was more abundant than the miRNA itself (for example, *miR-200, miR-2008, miR-219, miR-2011*) (**Figure S2**).

In summary, the sea urchin and sea star samples showed differences in the distribution of annotated small RNA classes, with the most striking difference being the relative higher enrichment of miRNAs in sea urchin embryos.

### Conservation of developmental miRNA gene expression in echinoderms

**Figure 3:**
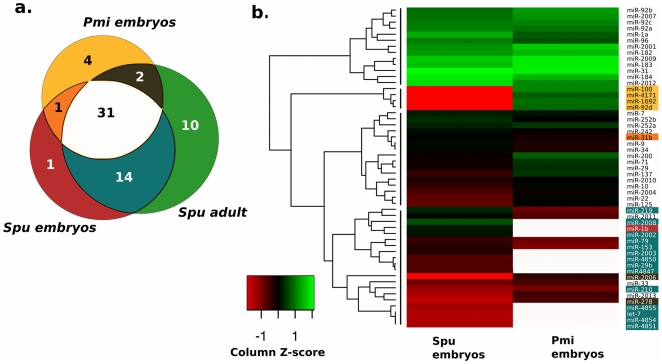
Comparison between sea urchin and sea star miRNAs. (**a**) Venn Diagram showing overlap between conserved miRNAs in sea urchin and sea embryos, and sea urchin adult (miRBase [34]). Only Illumina tags >2 reads were treated as potential true miRNAs. This figure does not include the miRNA* species. (**b**) Heat map showing the relative miRNA expression between sea urchin and sea star embryos (log_2_ transformed relative expression values). Average linkage clustering using Euclidean distance as the distance metric was used to generate the heat map (Methods). Since the genome sequence for sea star is unavailable, absence of certain miRNAs from the small RNA library in sea star, but its presence in sea urchin is treated as missing values for sea star. Missing values for sea star are indicated by the background color. Only miRNAs with zero reads are treated as missing values, whereas miRNAs with 1 or 2 reads are shown in the heat map.

We used sequence homology as well as information about the secondary stem-loop structure of precursor sequence to search for conserved and novel miRNAs in sea urchin and sea star embryonic libraries (see Methods). We found a total of 47 sea urchin and 38 sea star miRNAs mapping to known sequences in the miRBase registry (v. 17, April 2011) [34] (**Table 1**). **Fig. 3a** shows the overlap between miRNAs found expressed in the two embryonic libraries as well as adult sea urchins [26]. Overall, 53 miRNAs are expressed in one or both embryonic samples, whereas, 31 are expressed in sea urchin adults as well as in the embryonic stages of both species (**Fig. 3a**). This figure does not include the miRNA* species. When comparing miRNA expression between the two species, 25 are present in sea urchin only, 4 in sea star only (*miR-92d, miR-1692, miR-100, miR-4171*) and 34 in both species (**Fig. 3a**). The common hits are considered as putative candidates for phylum specific miRNAs. *miR-100* is considered a sea star specific miRNA in **Fig. 3** as it was absent in our sea urchin embryonic library and Wheeler *et al.* did not find this miRNA in the sea urchin adult by 454 sequencing [26]. Additionally, the current version of the sea urchin genome (version 2.1, UCSC Genome Browser [35]) lacks *miR-100* sequence as well. However, northern blot analysis previously showed that *miR-100* is present in sea urchin adult (coelomycytes and mesenchyme) [18]. It will be interesting to verify whether the adult tissue in sea urchin expresses it or not, thus, deciding its position as a species specific or phylum-conserved miRNA.

#### Novel miRNAs

We used miRDeep to identify potentially novel miRNAs in sea urchin [36] (we were not able to use miRDeep on the sea star dataset, because of the lack of the genomic sequence in this species.) Of the 11 novel predictions, 8 genes (5,183 reads) have seed sequences (positions 2–8) similar to known miRNAs in the registry (**Figure S3a**), while 3 are novel sequences with a total ∼400 reads. Each of the potentially novel sea urchin predictions is part of stem-loop genomic hairpins, characteristic of *Dicer* processing (**Figure S3**). The novel sea urchin predictions were also matched to sea star reads. Three out of the 11 predictions were found in sea star (**Table 1**). These three tags may therefore, represent echinoderm specific miRNAs. The other 10 tags may represent genes that have evolved after the divergence of the sea star and sea urchin lineages, although the sea star genome sequence is required before we make a definite assessment of this fact.

miRBase Release 17 (April 2011) [34] currently contains 64 sea urchin gene entries, all obtained from adult tissue by 454 sequencing [26], [27], including miRNA* species. No sea star miRNA genes are present in miRBase. Our embryonic libraries add 16 new sea urchin miRNA genes to this pool (2 conserved, 11 potentially novel and 3 miRNA*s); and 41 sea star miRNA genes (38 conserved, 3 potentially novel).

#### Comparison of miRNA genes expressed in embryos and adults

Most of the sea urchin miRNAs (45 out of 59) are expressed both in embryos (our dataset) and adults (miRBase registry) (**Fig. 3a**). However, twelve miRNAs are present in the adult sea urchin only, but not in the embryonic stages considered. These may correspond to adult-specific miRNAs with no role in development, or might have developmental roles outside of the embryonic stages considered for this study. On the other hand, *miR-31b* and *miR-1b* were found to be early development specific for the sea urchin, with no expression in the adult (**Fig. 3**). The most surprising result was *let-7* reads in the sea urchin embryos. Pasquinelli *et al.* [24], using northern blots, had shown that *S. purpuratus* embryos contain the *let-7* precursors, but not the mature *let-7* miRNA. We found 16 high-quality reads corresponding to this miRNA in our sample. We suspect that the relatively low abundance of this gene made it undetectable to northern blots. **Figure S4** shows the differences in sequence of *S. purpuratus* mature miRNAs between embryonic (Illumina sequencing) data and the adult 454 sequencing data. Most sequences are the same and few differences are seen at the 5′ or 3′ end. However, *miR-31b* shows a difference of one base at position 11.

There is no adult miRNA data for the *P. miniata* (PMI). However, Wheeler *et al*. [26] sequenced a species of sea star, *H. sanguinolenta* (HSN). On comparison of the PMI embryo data with the HSN adult data, 34 miRNAs were found in both species, 13 were found in HSN only and 8 were found in PMI only (**Figure S5**). Some changes are seen between the sequences of the same miRNA (indicated by *bold* in **Figure S5**) but most of these are at the 3′ end of the miRNA and could be due to different sequencing platforms or due to sequencing errors. The presence or absence of miRNAs between the two datasets might be due to different developmental stages, and might not represent species level changes.

In summary, we find that the pool of miRNAs is more or less conserved between embryonic and adult sea urchin. When we compared the developmentally expressed miRNAs between the two species we found that majority of them were conserved, although some relatively highly abundant miRNAs in sea urchin embryos did not have any reads in sea star embryos (for example, *miR-2008*) (**Fig. 3b**). The overall conservation of miRNA genes may imply that possible differences in miRNA function may be due to differences in their spatial expression or their expression levels.

### miRNA gene expression shows similar trends between the two echinoderm embryos

**Fig. 3b** shows a heat map corresponding to relative abundance of overlapping miRNAs between the sea urchin and sea star embryos. The miRNAs can be classified into 4 main groups based on their expression trends, (**1)** relatively high abundance in both species, **(2)** relatively high abundance in sea star embryos, but lower abundance in sea urchin embryos, **(3)** relatively high abundance in sea urchin embryos, but low abundance in sea star embryos, and **(4)** medium to low abundance in both species. Overall, we found that most miRNAs show similar patterns of expression in the two species. This indicates that the two echinoderms may share many features of their regulatory programs. However, some differences are also become apparent.

Out of the 14 highly expressed sea urchin miRNAs, 11 are also relatively highly expressed in sea star, which may indicate possible overlap in the post-transcriptional gene regulatory mechanisms. From the remaining three, two (*miR-183* and *miR-1a*) are of medium abundance in sea star, while *miR-2008* has a single read in sea star library (**Fig. 3b**). On the other hand, three highly expressed and one moderately expressed miRNA in sea star (*miR-1692*, *miR-100*, and *miR-92d*; and *miR-4171*, respectively) have no reads in the sea urchin library (**Fig. 3b**). These differentially expressed miRNAs are probably indicative of the differences between the two developmental programs. We note, however, that this is the first attempt to map the developmental post-transcriptional regulome in echinoderms, and spatial as well as temporal expression may vary even between the miRNAs that appear to be abundant in both species.

Since the embryonic libraries were a mixed population sample, northern blots of a few miRNAs in various early developmental stages of sea urchin and sea star embryos were used to confirm the presence of some conserved miRNAs (**Figure S6**). *miR-2009* was found in 1day, 2day and 3day old embryos in both species. *miR-31* and *miR-10* was found in all stages considered in sea urchin and sea star respectively. *miR-184* was only barely visible on the 3day old embryos of sea urchin with undetectable levels in 1day and 2day old embryos, and might be more development specific than the other miRNAs. However, the signal levels for sea star were undetectable. This might be due to the low sensitivity of the protocol (See Methods). It will be interesting to use whole mount in situ hybridization to compare the spatial and temporal patterns of these miRNAs.

### Evolution of miRNA sequences in the echinoderm animal lineage

**Figure 4:**
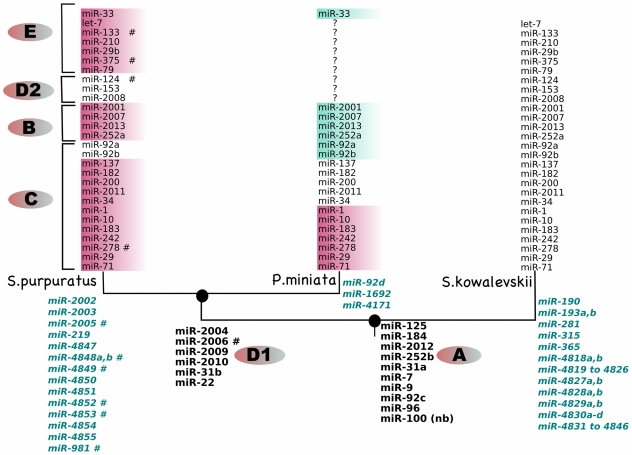
Phylogenetic comparison of sequence similarities between sea urchin, *S. purpuratus* and sea star, *P. miniata*. The hemichordate, *S. kowalevskii* has been used as the outgroup and the sequences in that species are used as the reference sequences. miRNA sequences in *S. purpuratus* or *P. miniata* that differ from the reference sequence are colored. Same color represents identical sequences. Absence of a miRNA from a species (represented by a blank) indicates absence of that miRNA from the reads and the registry. The miRNAs can be classified into 6 groups: (**A**) identical sequence and present in all three species; (**B**) present in all three species, but the sequence differences in all miRNAs; (**C**) present in all three species, but one or more species show mutations; (**D1**) identical sequence and present in *S. purpuratus* and *P. miniata*; (**D2**) identical sequence and present in *S. purpuratus* and *S. kowalevskii*; (**E**) present in two species with difference(s) in sequence; (**F**) the gene gained in a single species or lost in other two species. Group F is represented by the blue miRNAs at the node for the specific species *#: miRNA is in the registry but has ≤2 read frequency in the embryonic reads nb: miRNA was shown to be present in adult tissue by northern blot* [*18*] but is not present in registry. **: miR-2008 was found in late sea star embryos by whole mount in situ hybridization but not in early embryos ( ***Figure S8*** *)*.

miRNA families have been found in all analyzed animal lineages. It has been shown that evolutionary trends across metazoans show rare substitutions in mature miRNA sequence [26]. We found that about half of the miRNAs in sea urchin and sea star are identical in sequence, and the rest have acquired single or multiple mutations. All alignments between the three species are listed in **Figure S7**. Many of these differences are at the 3′ end of the miRNA, and represent the addition or loss of two or more bases. A mutation at the last base of the miRNA between two species is not treated as a change, as this might be a sequencing error and in any case it is not expected to affect the function of the mature miRNA. Differences at the 3′ end may be due to differences in the processing of the miRNA precursors between the two species. Striking differences are seen in abundant miRNAs such as, *miR-2001*, *miR-182, miR-183, miR-2007* and *miR-92b*, where the mutation(s) occurs in the middle of the sequence (**Figure S7**). **Fig. 4** shows the comparative analysis of mutations in miRNAs between the two echinoderms, using the hemichordate, acorn worm, *Saccoglossus kowalevskii* as an outgroup. The miRNAs can be grouped in several clusters based on the mutations across evolutionarily divergent species (**Fig. 4**). Only ten of the 28 miRNAs that are present in all three species (**Fig. 4**, categories A, B, and C) are identical in all of them; seven seem to have acquired mutations in the *S. kowalevskii* lineage (or in the echinoderm ancestor), five in the sea urchin lineage and only two in the sea star lineage. The remaining four miRNAs have differences in all three species (**Fig. 4**, category B). It will be very interesting to further investigate the effects of these mutations on the loss or gain of target sequences between the two echinoderms.

A very interesting observation was seen with *miR-2008*, which seemed to present in *S. purpuratus* and *S. kowalevskii*, but not in *P. miniata* based on our library data. Whole mount in situ hybridization on late stage sea star embryos showed that *miR-2008* is indeed present in sea star, but is not expressed in the early stage embryos considered for our library preparation (**Figure S8**).

We, thus, anticipate that our dataset will provide a rich source for future evolutionary studies, as both the miRNA and target sites may have evolved quite rapidly to facilitate new regulatory interactions.

## Methods

### Small RNA library preparation

Sea urchins and sea stars were collected by Marinus Scientific LLC in Southern California (http://www.marinusscientific.com/) and purchased by us. Total RNA was extracted from embryos at 24 h, 48 h and 72 h after fertilization using miRVana RNA isolation kit (Ambion). Embryo populations were combined, separately for each species, and the mixed population samples were sent for small RNA library preparation and sequencing to the Genomics & Microarray Facility at Wistar Institute, Philadelphia. Prior to library preparation, RNA quality was checked using the Bioanalyzer and was found to be very good with very little degradation (see **File S1 and Figure S1**).

Illumina adapters were ligated to the 5′ and 3′ ends of RNA, as described in the Illumina v1.5 protocol for small RNA sequencing samples. Small RNA molecules were size selected (**Figure S1**), and RT-PCR amplification was used to generate the cDNA libraries for both species. The 36 bp run on the Illumina Genome Analyzer (Illumina, Inc.) was used for sequencing these cDNAs.

### Computational analysis procedure and pipeline

**Figure 5:**
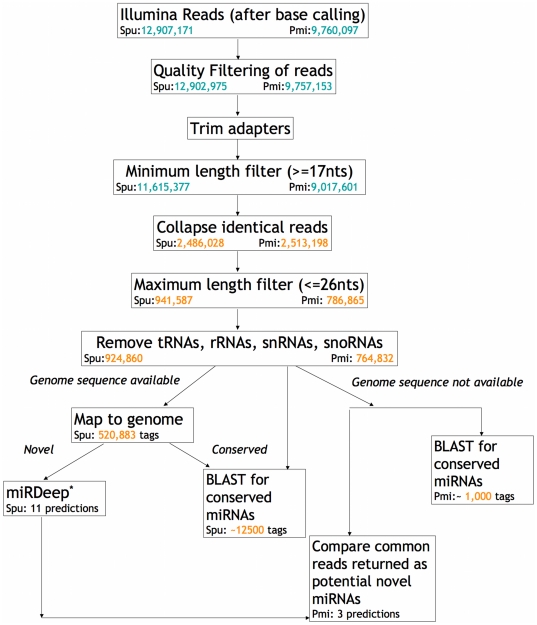
Computational pipeline for analysis of deep sequencing libraries for discovery of small non-coding RNAs. Illumina reads undergo numerous filtering steps based on quality and length. The pipeline has two branches: for a species with genome sequence, and for a species without a sequenced genome, but a closely related sequenced species. *Spu: Strongylocentrotus purpuratus; Pmi: Patiria miniata*. miRDeep [36]; BLAST [38]. *Green color*: Reads *Orange*: Tags.

Base calling was performed by the Bioinformatics facility at Wistar Institute. The resulting sequences were subjected to our computational pipeline (**Fig. 5**), which consists of a number of in-house made scripts. First, we perform quality filtering by converting the Illumina quality codes for each base to its Phred quality score, and trimming the low quality 3′ ends of the reads. A cut-off of 20 was selected based on the histogram of qualities for all reads (data not shown). 3′ adapters were trimmed using the *novoalign* program (www.novocraft.com). This program uses ungapped semi-global alignment of adapter sequence against the read using a weight matrix from read and base qualities, and trimming is performed from start of the optimum alignment. 5′ adapter sequence was trimmed based on perfect sequence match of more than or equal to 10 nts at the 5′ end. All reads shorter than 17 nts or longer than 26 nts were excluded from further analysis except when it is noted otherwise. The remaining reads with 100% sequence identity and length difference of 2 nts or less were collapsed to produce “*tags*” of genes and calculate their expression as number of independent reads each tag has. At this stage, tRNAs, rRNAs, snRNAs and snoRNAs are removed based on sequence identity to known genes. Also, similarity to known miRNAs is used to identify evolutionary conserved miRNAs. If a genome is available (i.e., sea urchin, in our case) the reads are mapped to the genome and novel miRNA genes are discovered using miRDeep [36].

Sea urchin tRNA sequences were obtained from UCSC (http://gtrnadb.ucsc.edu/) and snRNA and snoRNA sequences from GenBank [37]. rRNA sequences were gathered from a variety of sources for three sea urchin species (*S. purpuratus, P. lividus, L. variegatus*), including UCSC genome browser [35] and EBI databases (http://www.ebi.ac.uk/Databases/). Since there is no tRNA, snoRNA or snRNA data publicly available for the sea star, the sequences from sea urchin were used for the search in sea star. Due to the highly diverse nature of piRNAs and the fact that a large number of them represent lowly expressed genes, we decided to exclude piRNAs from our analysis. For sequence similarity match we used BLAST [38]. The parameters used to map miRNAs to Illumina reads were “-e 0.01 -p 100 -W 8”. For mapping reads to the genome and other conserved sequences, parameters used were “-W 12 -p 80”. All hits with length less than 85% of the length of the query sequence were ignored. mRNA sequences for the sea urchin and sea star were compiled using NCBI predicted genes [37] and the SpBase (http://spbase.org) database [39] was also used for *S. purpuratus*.

The computational pipeline to analyze Illumina reads is available at http://www.benoslab.pitt.edu/services.html.

### Hierarchical clustering of gene expression values

The relative abundance of each miRNA in each sample was log2 transformed for better visualization of the data. Average linkage hierarchical clustering was performed using Euclidean distance as the distance metric. The distance between two clusters X and Y is given by:
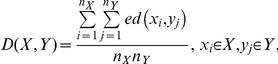
where 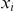 is the vector of log2 transformed relative abundances of miRNA *i*, 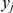 is the vector of log2 transformed relative abundances of miRNA *j*, 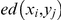 is the Euclidean distance between 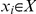 and 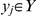, 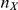 is the number of samples in cluster 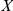, 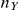 is the number of samples in cluster 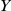.

### Whole mount in situ hybridization

We followed our lab protocol [40] except we used an antisense 3′ DIG labeled locked nucleic acid (LNA) probe (Exiqon Inc.) at concentrations of between 2pmol to 4pmol per 100 ul of hybridization solution and at 47°C as recommended by the supplier.

### Northern Blots

Total RNA was extracted from sea urchin and starfish embryos using the miRVana kit by Ambion. Standard northern blot protocols were performed using 10–15 µg of total RNA and antisense miRNAs, starfireTM (IDT) α-P32 oligonucleotide labeled probe. A 10 nt to 100 nt size ladder was used (Decade, Ambion) to estimate size.

## Supporting Information

File S1 **RNA quality.**
(DOC)

Figure S1 **The RNA quality was checked using the BioAnalyzer before (a,b) and after (c) adapter ligation.** (**a**) Distribution of lengths of the RNA sample from sea urchin before adapters were ligated. The first peak (∼20–25 nt) corresponds to the small RNA population. (**b**) Length distribution of sea star RNA sample before adapter ligation. (**c**) The adapter-ligated RNA was run on a gel and size-selected for small RNAs.
(PNG)

Figure S2 Reads for mature miRNA and miRNA* in UCSC genome browser for the sea urchin. Reads (logarithm scale) for miRNA and miRNA* for cases in which the miRNA* is more abundant than miRNA.
(PNG)

Figure S3 **Stem-loop structures of the novel miRNA miRDeep (1) predictions in sea urchin.** (a) miRNAs that share their seeds with known miRNAs. The temporary labels are the names of miRNA (b) Precursors of novel miRNAs without any seed conservation.
(PNG)

Figure S4 **Comparison of mature miRNA sequences between** ***S. purpuratus*** **adult (2) and embryonic data.** Differences are highlighted in bold. **E:** Embryonic data from Illumina platform; **A:** Adult data from 454 sequencing platform.
(PDF)

Figure S5 **Comparison of mature miRNA sequences between** ***H.sanguinolenta*** **adult data (2) and** ***P.miniata*** **embryonic data.** Differences are highlighted in bold. *Pmi: P. miniata; Hsn: H. sanguinolenta.*
(PDF)

Figure S6 **Northern Blot showing the expression of a few conserved miRNAs in** ***S. purpuratus*** **(sea urchin) and** ***P. miniata*** **(sea star) embryos.** 5S rRNA is used as the loading control while *miR-124* is used as the negative control.
(TIF)

Figure S7 **Alignment of mature miRNA sequences in two echinoderms and a hemichordate reference species.** ***spu - S. purpuratus; pmi - P. miniata; sko - S. kowalevskii.***
(PDF)

Figure S8 **Whole mount in situ hybridization of** ***P. miniata*** **embryos using LNA probes antisense to** ***miR-2008*** **.** Blastula and gastrula stages do not show any expression for this miRNA, consistent with the embryonic small RNA library. However, we see expression of *miR-2008* in late stage larvae.
(PNG)
