# Supplementary material for: RNA Deep Sequencing Reveals Differential MicroRNA Expression during Development of Sea Urchin and Sea Star
Source: PLoS One. 2011 Dec 28;6(12):e29217. doi: 10.1371/journal.pone.0029217 (PMC3247247; doi:10.1371/journal.pone.0029217)
Supplement: File S1 — RNA quality. (DOC) [file pone.0029217.s001.doc]

Supporting Information for:

"RNA deep sequencing reveals differential microRNA expression during development of sea urchin and sea star "

^12,*^, Panayiotis V. Benos^3,*^

^1^ Lane Center for Computational Biology, Carnegie Mellon University, Pittsburgh, Pennsylvania, USA

^2^ Department of Biological Sciences, Carnegie Mellon University, Pittsburgh, Pennsylvania, USA

^3^ Department of Computational and Systems Biology, University of Pittsburgh, Pittsburgh, Pennsylvania, USA

Email: SK ([sskadri@andrew.cmu.edu](mailto:sskadri@andrew.cmu.edu)), VH ([vhinman@cmu.edu](mailto:vhinman@cmu.edu)), PVB ([benos@pitt.edu](mailto:benos@pitt.edu))

* Corresponding author

**RNA quality:**

The Bioanalyzer profiles for the total RNA of the sea urchin and sea star embryos are presented in **Suppl. Fig. S1a** and **Suppl. Fig. S1b**. These results indicate good RNA quality. There are three prominent peaks – the smallest peak corresponds to 5s rRNA and small RNAs in the sample, while the other peaks correspond to 18s and 28s rRNAs respectively. Although the rRNA profile is not a definitive indication of RNA integrity, the profiles show that smaller RNAs are not hidden by degraded products of different sizes. The gel in **Suppl. Fig S1c** shows the RNA after adapter ligation. The highlighted band was excised to run on the Illumina Genome Analyzer (Illumina, Inc.).

**REFERENCES**

1. Friedlander MR, Chen W, Adamidi C, Maaskola J, Einspanier R, Knespel S, Rajewsky N. Discovering microRNAs from deep sequencing data using miRDeep. Nature biotechnology2008 Apr;26(4):407-15.

2. Wheeler BM, Heimberg AM, Moy VN, Sperling EA, Holstein TW, Heber S, Peterson KJ. The deep evolution of metazoan microRNAs. Evol Dev2009 Jan-Feb;11(1):50-68.
