# Supplementary figures and images for: RNA Deep Sequencing Reveals Differential MicroRNA Expression during Development of Sea Urchin and Sea Star
Source: PLoS One. 2011 Dec 28;6(12):e29217. doi: 10.1371/journal.pone.0029217 (PMC3247247; doi:10.1371/journal.pone.0029217)

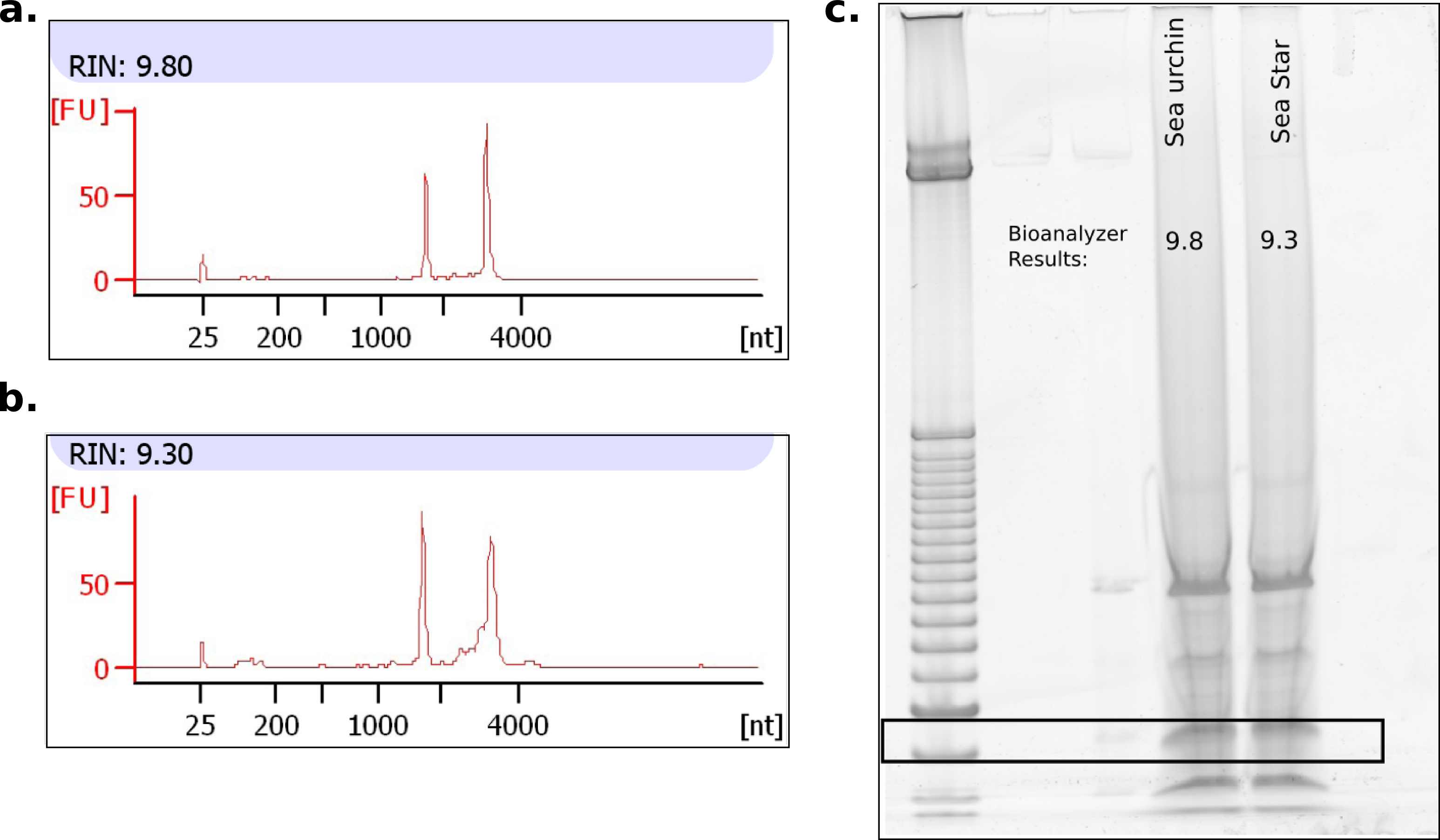

Supplement: Figure S1 — The RNA quality was checked using the BioAnalyzer before (a,b) and after (c) adapter ligation. (a) Distribution of lengths of the RNA sample from sea urchin before adapters were ligated. The first peak (∼20–25 nt) corresponds to the small RNA population. (b) Length distribution of sea star RNA sample before adapter ligation. (c) The adapter-ligated RNA was run on a gel and size-selected for small RNAs. (PNG) [file pone.0029217.s002.png]

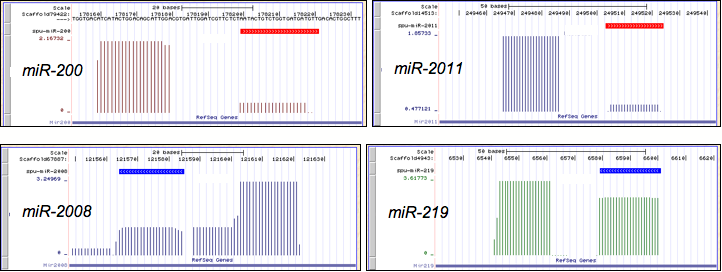

Supplement: Figure S2 — Reads for mature miRNA and miRNA* in UCSC genome browser for the sea urchin. Reads (logarithm scale) for miRNA and miRNA* for cases in which the miRNA* is more abundant than miRNA. (PNG) [file pone.0029217.s003.png]

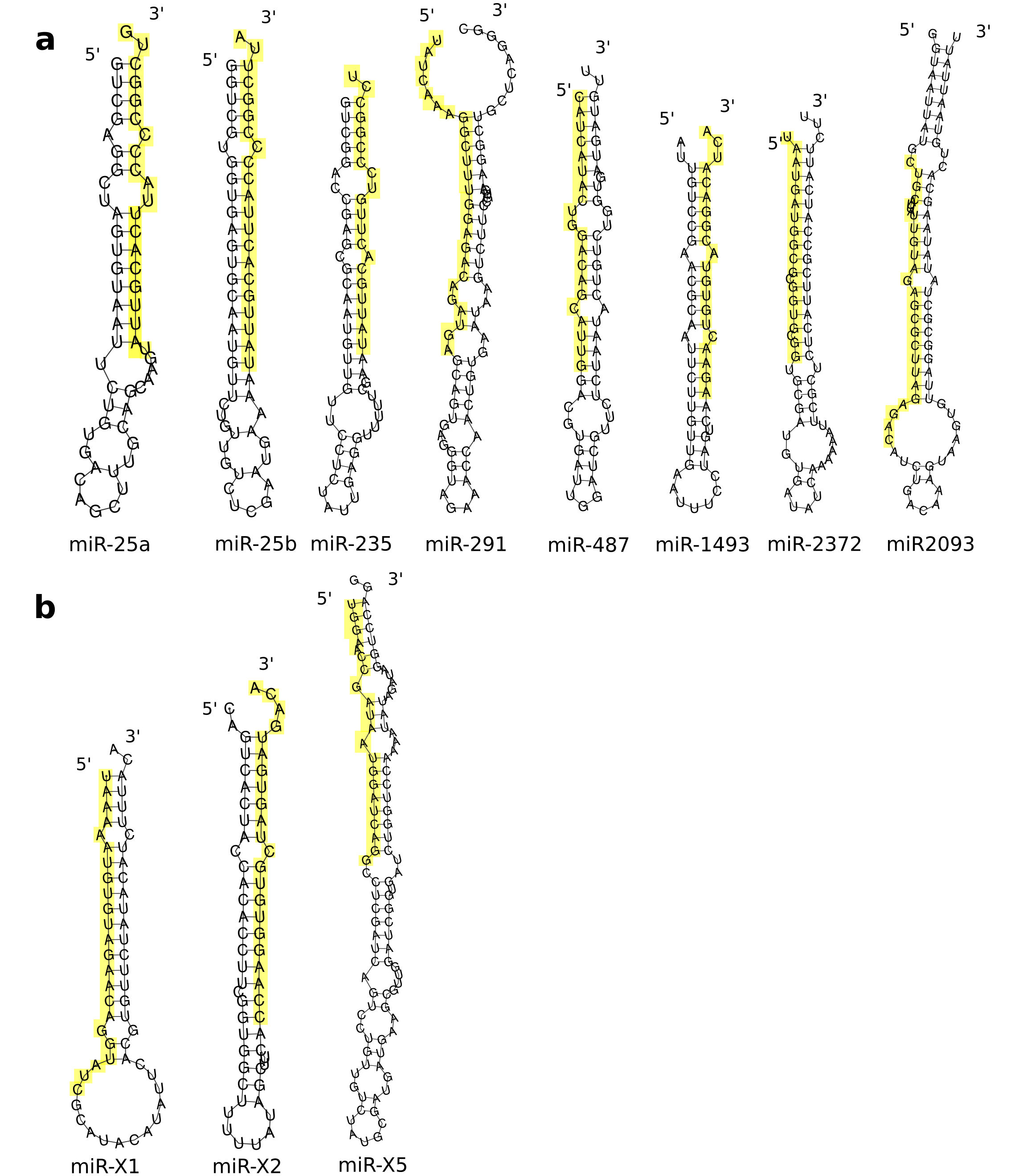

Supplement: Figure S3 — Stem-loop structures of the novel miRNA miRDeep (1) predictions in sea urchin. (a) miRNAs that share their seeds with known miRNAs. The temporary labels are the names of miRNA (b) Precursors of novel miRNAs without any seed conservation. (PNG) [file pone.0029217.s004.png]

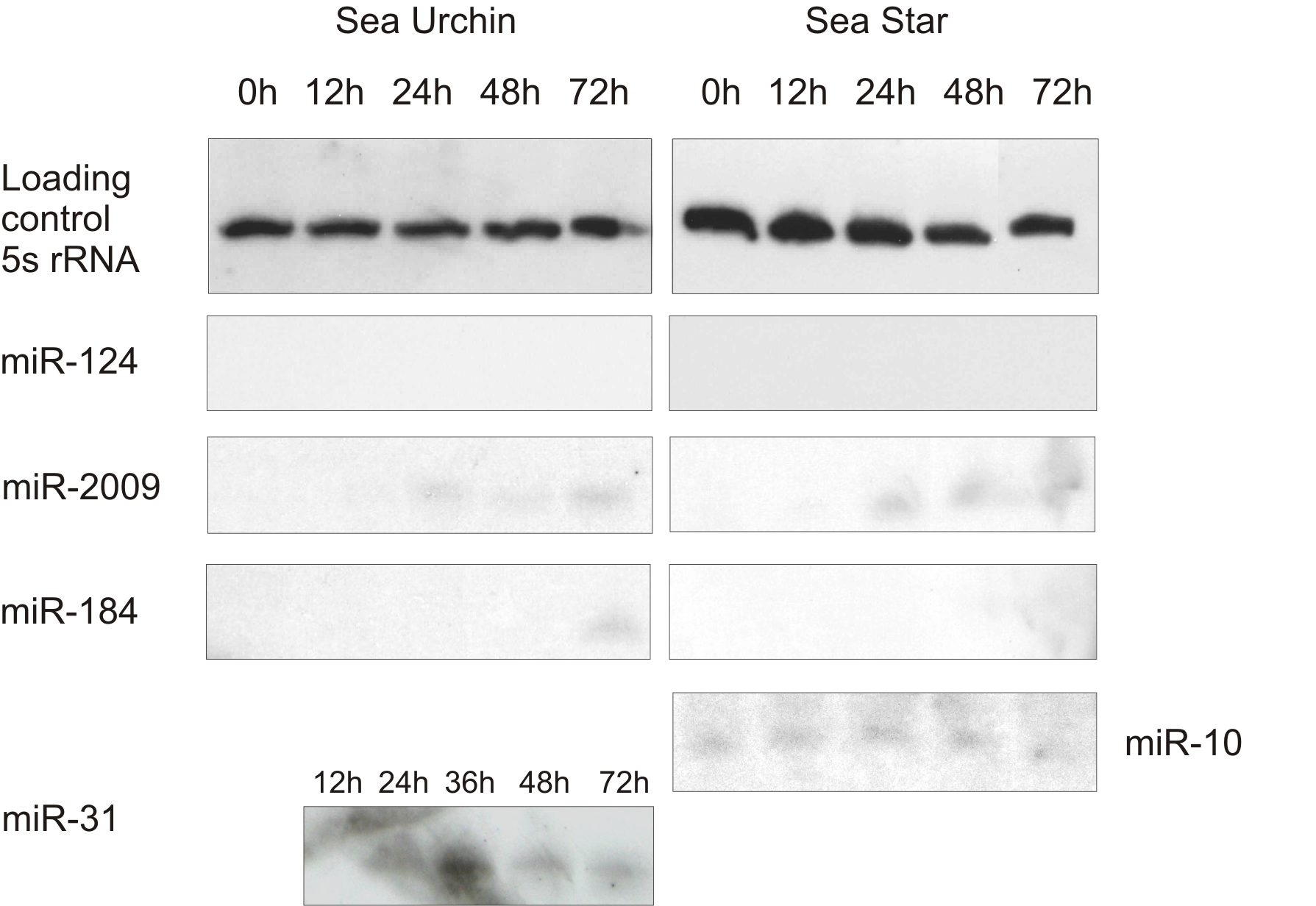

Supplement: Figure S6 — Northern Blot showing the expression of a few conserved miRNAs in S. purpuratus (sea urchin) and P. miniata (sea star) embryos. 5S rRNA is used as the loading control while miR-124 is used as the negative control. (TIF) [file pone.0029217.s007.tif]

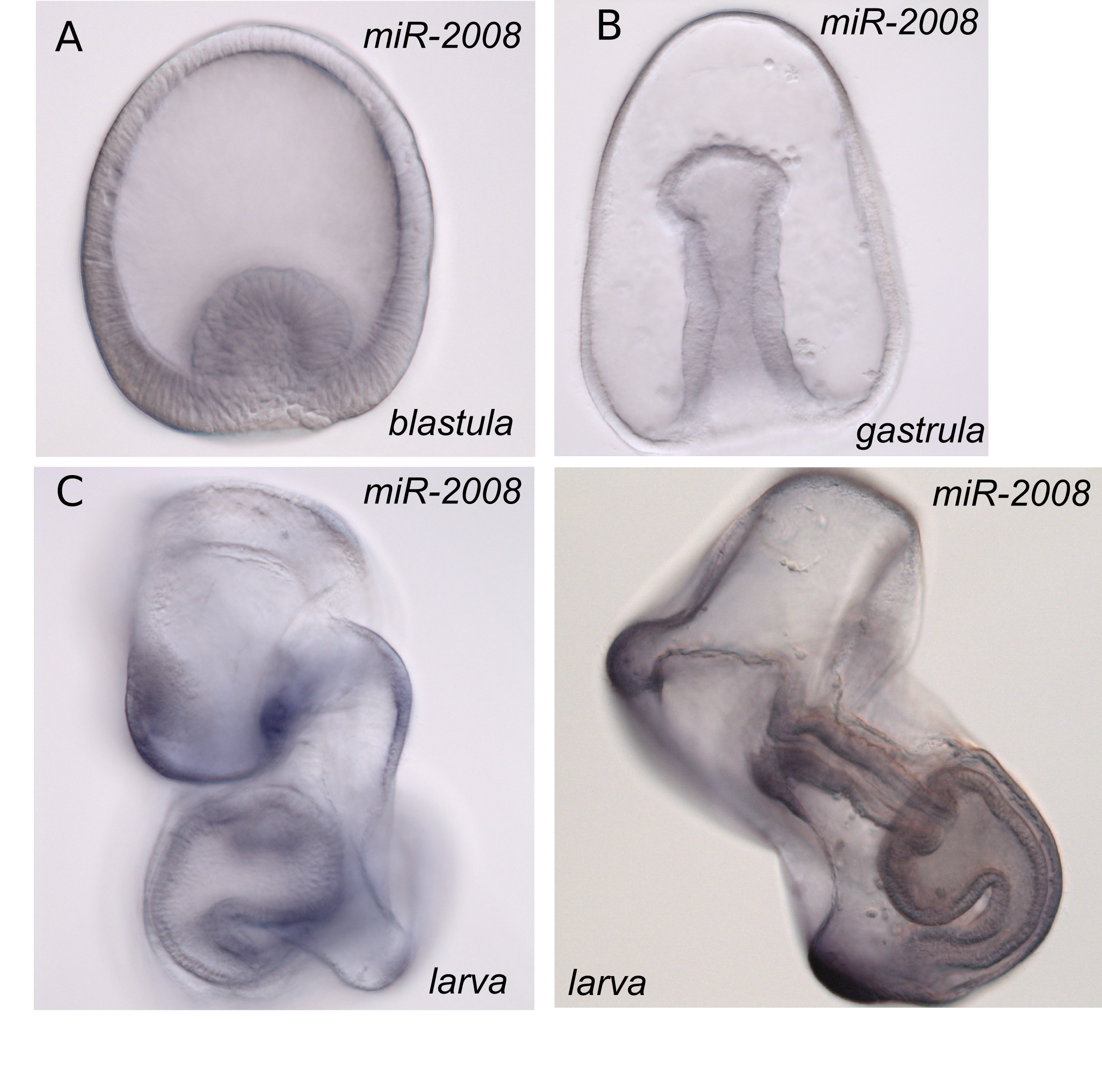

Supplement: Figure S8 — Whole mount in situ hybridization of P. miniata embryos using LNA probes antisense to miR-2008 . Blastula and gastrula stages do not show any expression for this miRNA, consistent with the embryonic small RNA library. However, we see expression of miR-2008 in late stage larvae. (PNG) [file pone.0029217.s009.png]
