# Supplementary material for: RNA Deep Sequencing Reveals Differential MicroRNA Expression during Development of Sea Urchin and Sea Star
Source: PLoS One. 2011 Dec 28;6(12):e29217. doi: 10.1371/journal.pone.0029217 (PMC3247247; doi:10.1371/journal.pone.0029217)
Supplement: Figure S4 — Comparison of mature miRNA sequences between S. purpuratus adult (2) and embryonic data. Differences are highlighted in bold. E: Embryonic data from Illumina platform; A: Adult data from 454 sequencing platform. (PDF) [file pone.0029217.s005.pdf]

**miRNA**	**Sequence**
spu-let-7	E: TGAGGTAGTAGGTTATATAGTT<br>A: TGAGGTAGTAGGTTATATAGTT
spu-miR-1	E: TGGAATGTAAAGAAGTATGTAT<br>A: TGGAATGTAAAGAAGTATGTAT
spu-miR-1b	E: TGGAATGTAAAGAAGTATGTAC
spu-miR-10	E: AACCCTGTAGATCCGAATTTGTG<br>A: AACCCTGTAGATCCGAATTTGTG
spu-miR-125	E: TCCCTGAGACCCTAACTTGTGA<br>A: TCCCTGAGACCCTAACTTGTGA
spu-miR-137	E: **T**TATTGCTTGAGAATACACGTA-<br>A: -TATTGCTTGAGAATACACGTA**G**
spu-miR-153	E: TTGCATAGTCACAAAAGTGATT<br>A: TTGCATAGTCACAAAAGTGATT
spu-miR-182	E: TTTGGCAATTGATAGAATTCACACT<br>A: TTTGGCAATTGATAGAATTCACACT
spu-miR-183	E: TATGGCACTATAGAATTCACTG<br>A: TATGGCACTATAGAATTCACTG
spu-miR-184	E: TGGACGGAGAACTGATAAGGGC<br>A: TGGACGGAGAACTGATAAGGGC
spu-miR-200	E: TAATACTGTCTGGTGATGATGTT<br>A: TAATACTGTCTGGTGATGATGTT
spu-miR-2001	E: ATGTGACCGATATAATGGGCAT<br>A: ATGTGACCGATATAATGGGCAT
spu-miR-2002	E: TGAATACATCTGCTGGTTTTTAT<br>A: TGAATACATCTGCTGGTTTTTAT
spu-miR-2003	E: AACCCGTAAGGTCTTAACTTGTG<br>A: AACCCGTAAGGTCTTAACTTGTG
spu-miR-2004	E: TCACACACAACCACAGGAAGTT<br>A: TCACACACAACCACAGGAAGTT
spu-miR-2007	E: TATTTCAGGCAGTATACTGGTAA<br>A: TATTTCAGGCAGTATACTGGTAA
spu-miR-2008	E: ATCAGCCTCGCTGTCAATACG**A**<br>A: ATCAGCCTCGCTGTCAATACG-
spu-miR-2009	E: TGAGTTGTCCCACAAAGAACAC-<br>A: TGAGTTGTCCCACAAAGAACAC**A**
spu-miR-2010	E: TTACTGTTGATGTCAGCCCCTT<br>A: TTACTGTTGATGTCAGCCCCTT
spu-miR-2011	E: ACCAAGGTGTGCTAGTGATGAC<br>A: ACCAAGGTGTGCTAGTGATGAC
spu-miR-2012	E: TAGTACTGGCATATGGACATTG<br>A: TAGTACTGGCATATGGACATTG
spu-miR-2013	E: TGCAGCATGATGTAGTGGTGT**A**<br>A: TGCAGCATGATGTAGTGGTGT-
spu-miR-210	E: TTGTGCGTGCGACAGCGACTGA<br>A: TTGTGCGTGCGACAGCGACTGA
spu-miR-219	E: TGATTGTCCGAACGCAATTCTTG<br>A: TGATTGTCCGAACGCAATTCTTG
spu-miR-22	E: **T**CAGCTGCCCGGTGAAGTGTATA<br>A: -CAGCTGCCCGGTGAAGTGTATA
spu-miR-242	E: TTGCGTAGGCGTTGTGCACAGT<br>A: TTGCGTAGGCGTTGTGCACAGT
spu-miR-252a	E: CTAAGTACTAGTGCCGTAGGTT<br>A: CTAAGTACTAGTGCCGTAGGTT
spu-miR-252b	E: CTAAGTAGTAGTGCCGCAGGTA

	A: CTAAGTAGTAGTGCCGCAGGTA
spu-miR-29	E: AAGCACCAGTTGAAATCAGAGC<br>A: AAGCACCAGTTGAAATCAGAGC
spu-miR-29b	E: TAGCACCATGAGAAAGCAGTAT<br>A: TAGCACCATGAGAAAGCAGTAT
spu-miR-31a	E: AGGCAAGATGTTGGCATAGCT**G**<br>A: AGGCAAGATGTTGGCATAGCT-
spu-miR-31b	E: AGGCAAGATGCTGGCATAGCT
spu-miR-33	E: GTGCATTGTCGTTGCATTGCAT<br>A: GTGCATTGTCGTTGCATTGCAT
spu-miR-34	E: CGGCAGTGTAGTTAGCTGGTTG<br>A: CGGCAGTGTAGTTAGCTGGTTG
spu-miR-4847	E: TAATGATGGCGCGGTGCGGTGC<br>A: TAATGATGGCGCGGTGCGGTGC
spu-miR-4850	E: TTATCATGACTGTAAACAGGAGG<br>A: TTATCATGACTGTAAACAGGAGG
spu-miR-4851	E: TGATTACTTGCTTTGGAGTTCTT<br>A: TGATTACTTGCTTTGGAGTTCTT
spu-miR-4854	E: TGTTGCAGTGACGACTTCGCGC<br>A: TGTTGCAGTGACGACTTCGCGC
spu-miR-4855	E: TGTGTAACATCTCATTCAGTGGGT<br>A: TGTGTAACATCTCATTCAGTGGGT
spu-miR-7	E: TGGAAGACTAGTGATTTTGTTGT<br>A: TGGAAGACTAGTGATTTTGTTGT
spu-miR-71	E: TGAAAGACATGGGTAGTGAGATT<br>A: TGAAAGACATGGGTAGTGAGATT
spu-miR-79	E: ATAAAGCTAGGTTACCAAAGAT**A**<br>A: ATAAAGCTAGGTTACCAAAGAT-
spu-miR-9	E: TCTTTGGTTATCTAGCTGTATG<br>A: TCTTTGGTTATCTAGCTGTATG
spu-miR-92a	E: TATTGCACTTGTCCCGGCCTAC-<br>A: TATTGCACTTGTCCCGGCCTAC**T**
spu-miR-92b	E: TATTGCACTTGTCCCGGCCTGC<br>A: TATTGCACTTGTCCCGGCCTGC
spu-miR-92c	E: TATTGCACTCGTCCCGGCCTGC<br>A: TATTGCACTCGTCCCGGCCTGC
spu-miR-96	E: TTTGGCACTAGCACATTTTGC<br>A: TTTGGCACTAGCACATTTTGC

E: Embryo data from Illumina sequencing
A: Adult data from miRBase
