# Supplementary material for: RNA Deep Sequencing Reveals Differential MicroRNA Expression during Development of Sea Urchin and Sea Star
Source: PLoS One. 2011 Dec 28;6(12):e29217. doi: 10.1371/journal.pone.0029217 (PMC3247247; doi:10.1371/journal.pone.0029217)
Supplement: Figure S5 — Comparison of mature miRNA sequences between H.sanguinolenta adult data (2) and P.miniata embryonic data. Differences are highlighted in bold. Pmi: P. miniata; Hsn: H. sanguinolenta. (PDF) [file pone.0029217.s006.pdf]

miR-1	Pmi TGGAATGTAAAGAAGTATGTAT<br>Hsn TGGAATGTAAAGAAGTATGTAT
miR-7	Pmi TGGAAGACTAGTGATTTTGTTGT<br>Hsn TGGAAGACTAGTGATTTTGTTGT
miR-8, 141, 200	Pmi TAATACTGTCTGGTAATGATGTT<br>Hsn<br>R1 TAATACTGTCTGGTAATGATGT-
miR-9	Pmi TCTTTGGTTATCTAGCTGTATGA<br>Hsn<br>R1 TCTTTGGTTATCTAGCTGTATGA
miR-10	Pmi AACCCTGTAGATCCGAATTTGT**G**<br>Hsn<br>R1 AACCCTGTAGATCCGAATTTGT-
miR-22, 745, 980	Pmi TCAGCTGCCCGGTGAAGTGTAG<br>Hsn TCAGCTGCCCGGTGAAGTGTAG
miR-29, 83, 285	Pmi AAGCACCAGTTGAAATCAGAGC<br>Hsn<br>R1 AAGCACCAGTTGAAATCAGAGC
miR-31	Pmi<br>31a AGGCAAGATGTTGGCATAGCTG<br>31b AGGCAAGATG**C**TGGCATAGCTG<br>Hsn<br>R1 AGGCAAGATGTTGGCATAGCT-
miR-33	Pmi GTGCATTGTAGTTGCATTGCAT<br>Hsn GTGCATTGTAGTTGCATTGCAT
miR-34	Pmi TGGCAGTGTGGTTAGCTGGTTG<br>Hsn TGGCAGTGTGGTTAGCTGGTTG
miR-71	Pmi TGAAAGACATGGGTAGTGAGAT<br>Hsn<br>R1 TGAAAGACATGGGTAGTGAGAT
miR-79	Pmi ATAAAGCTAGGTTACCAAAGATA<br>Hsn -TAAAGCTAGGTTACCAAAGAT-
miR-92	Pmi<br>92a TATTGCACTTGTCC**C**CGGCC**A**GC<br>92b TATTGCACTTGTCTCGGCCTGC<br>92c TATTGCACT**C**GTC**C**CGGCCTGC<br>92d TATTGCACT**C**GTC**C**CGGCCT**AG**<br>Hsn TATTGCACTTGTCTCGGCCTGC
miR-96	Pmi TTTGGCACTAGCACATTTTGC-<br>Hsn TTTGGCACTAGCACATTTTGCT
miR-100	Pmi AACCCGTAGATCCGAACTTGT<br>Hsn AACCCGTAGATCCGAATTTGT
miR-124	Hsn TAAGGCACGCGGTGAATGCCA
miR-125	Pmi TCCCTGAGACCCTAACTTGTGA<br>Hsn<br>R1 TCCCTGAGACCCTAACTTGTGA
miR-133	Hsn TTTGGTCCCCTTCAACCAGCCGT
miR-137	Pmi TATTGCTTGAGAATACACGTAG<br>Hsn TATTGCTTGAGAATACACGTAG
miR-153	Hsn TTGCATAGTCACAAAAGTGATT
miR-1692	Pmi TGTAGCTCAGTTGGTAGAG
miR-182, 263b	Pmi TTTGGCAATAGATAGAATTCACA<br>Hsn TTTGGCAATAGATAGAATTCAC-
miR-183	Pmi TATGGCACTGTAGAATTCACT

miR-184	Pmi TGGACGGAGAACTGATAAGGGC<br>Hsn<br>R1 TGGACGGAGAACTGATAAGGGC
miR-190	Hsn TGATATGTTTGGTTTTGGGTTG
miR-193	Hsn TACTGGCCAGCACAATCCCAGA
miR-210	Hsn TTGTGCGTGCGACAGCGACTGA
miR-219	Pmi TGATTGTCCAAACGCAATTCT
miR-242	Pmi TTGCGTAGGCGTTGTGCACAGT<br>Hsn TTGCGTAGGCGTTGTGCACAGT
miR-252a	Hsn CTAAGTACTAGTGCCGCAGTT-<br>Pmi CTAAGTACTAGTGCCGCAGTT**G**
miR-252b	Pmi CTAAGTAGTAGTGCCGCAGGTA<br>Hsn CTAAGTAGTAGTGCCGCAGGTA
miR-278	Pmi TCGGTGGGACTTTCGTTCGAT**T**<br>Hsn TCGGTGGGACTTTCGTTCGAT-
miR-365	Hsn TAATGCCCTGTGAAATCCCAAA
miR-375	Hsn TTTGTTCGTTCGGCTCGCGTCA
miR-2001	Pmi ATGTGACCGTTACAATGGGCAT<br>Hsn ATGTGACCGTTACAATGGGCAT
miR-2002	Hsn<br>G T-GAATACATCTGCTGGTTCCAT<br>R1 T-GAATACATCTGCTGGTTCCAT<br>R2 TAGAATACATCTGCTGGTTCCAT
miR-2003	Hsn AACCCGTAAGATCCGAACTTGT
miR-2004	Pmi TCACACACAACCACAGGAAGT**T**<br>Hsn TCACACACAACCACAGGAAGT-
miR-2005	Hsn AGTCCAATAGGGAGGGCATCGC
miR-2006-3p	Pmi GAGCACACTTGGTAGCGGTGCC<br>Hsn GAGCACACTTGGTAGCGGTGCC
miR-2006-5p	Hsn CATCGCCACCAGGTGTACTTCA-
miR-2007	Hsn TATTTCAGGCAGTATACTGGTAA<br>Pmi TATTTCAGGC**G**GTATACTGGTAA
miR-2008-3p	Pmi ATCAGCCTCGCTGTCAATAC**GA**<br>Hsn<br>R2 ATCAGCCTCGCTGTCAATAC--
miR-2009	Pmi TGAGTTGTCCCACAAAGAACAC<br>Hsn TGAGTTGTCCCACAAAGAACAC
miR-2010	Pmi TTACTGTTGATGTCAGCCCCT**C**<br>Hsn TTACTGTTGATGTCAGCCCCT-
miR-2011	Pmi ACCAAGGTGTGTTAGTGATGAC<br>Hsn<br>R2 ACCAAGGTGTGTTAGTGATGAC
miR-2012	Pmi TAGTACTGGCATATGGACATT-<br>Hsn TAGTACTGGCATATGGACATT**C**
miR-2013	Pmi TGCAGCATGATGTAGTGGTG**A**<br>Hsn TGCAGCATGATGTAGTGGTG-
miR-4171	Pmi TGACTCTCTTAAGGTAGCC
let-7	Hsn<br>R1 T-GAGGTAGTCGGTTGTAAAGA<br>R2 TAGAGGTAGTCGGTTGTAAAGA<br>R3 T-GATGTAGTCGGTTGTAAAGA
