# Supplementary material for: RNA Deep Sequencing Reveals Differential MicroRNA Expression during Development of Sea Urchin and Sea Star
Source: PLoS One. 2011 Dec 28;6(12):e29217. doi: 10.1371/journal.pone.0029217 (PMC3247247; doi:10.1371/journal.pone.0029217)
Supplement: Figure S7 — Alignment of mature miRNA sequences in two echinoderms and a hemichordate reference species. spu - S. purpuratus; pmi - P. miniata; sko - S. kowalevskii. (PDF) [file pone.0029217.s008.pdf]

```
spu-let-7         TGAGGTAGTAGGTTATATAGTT 22
sko-let-7         TGAGGTAGTAGGTTGTATAGTT 22
                  ************** *******

spu-miR-1         TGGAATGTAAAGAAGTATGTAT 22
pmi-miR-1         TGGAATGTAAAGAAGTATGTAT 22
sko-miR-1         TGGAATGTAATGAAGTATGTAT 22
                  ********** ***********

spu-miR-1b        TGGAATGTAAAGAAGTATGTAC 22
sko-miR-1b        TGGAATGTAATGAAGTATGTAT 22
                  ********** **********

spu-miR-10        AACCCTGTAGATCCGAATTTGTG 23
pmi-miR-10        AACCCTGTAGATCCGAATTTGTG 23
sko-miR-10        TACCCTGTAGATCCGAATTTGTG 23
                   **********************

pmi-miR-100       AACCCGTAGATCCGAACTTGT- 21
sko-miR-100       AACCCGTAGATCCGAACTTGTG 22
                  *********************

spu-miR-124       TAAGGCACGCGGTGAATGCCA- 21
sko-miR-124       TAAGGCACGCGGTGAATGCCAA 22
                  *********************

spu-miR-125       TCCCTGAGACCCTAACTTGTGA 22
pmi-miR-125       TCCCTGAGACCCTAACTTGTGA 22
sko-miR-125       TCCCTGAGACCCTAACTTGTGA 22
                  **********************

spu-miR-133       TTTGGTCCCCTTCAACCAGCCGT 23
sko-miR-133       -TTGGTCCCCTTCAACCAGCTGT 22
                   ******************* **

spu-miR-137       TTATTGCTTGAGAATACACGT—- 21
pmi-miR-137       -TATTGCTTGAGAATACACGTAG 22
sko-miR-137       -TATTGCTTGAGAATACACGTAG 22
                   ********************

spu-miR-153       TTGCATAGTCACAAAAGTGATT 22
sko-miR-153       TTGCATAGTCACAAAAGTGATT 22
                  **********************

pmi-miR-1692      TGTAGCTCAGTTGGTAGAG 19

spu-miR-182       TTTGGCAATTGATAGAATTCACACT 25
pmi-miR-182       TTTGGCAATAGATAGAATTCACA-- 23
sko-miR-182       TTTGGCAATAGATAGAATTCACA-- 23
                  ********* *************

spu-miR-183       TATGGCACTATA-GAATTCACTG 22
pmi-miR-183       TATGGCACTGTA-GAATTCACT- 21
sko-miR-183       AATGGCACTGTATGAATTCACTG 23
                   ******** ** **********
```

```
spu-miR-184       TGGACGGAGAACTGATAAGGGC 22
pmi-miR-184       TGGACGGAGAACTGATAAGGGC 22
sko-miR-184       TGGACGGAGAACTGATAAGGGC 22
                  **********************

spu-miR-200       TAATACTGTCTGGTGATGATGTT 23
pmi-miR-200       TAATACTGTCTGGTAATGATGTT 23
sko-miR-200       TAATACTGTCTGGTAATGATGTT 23
                  ************** ********

spu-miR-2001      ATGTGACCGATATAATGGGCAT 22
pmi-miR-2001      ATGTGACCGTTACAATGGGCAT 22
sko-miR-2001      TTGTGACCGTTATAATGGGCAT 22
                   ******** ** *********

spu-miR-2002      TGAATACATCTGCTGGTTTTTAT 23

spu-miR-2003      AACCCGTAAGGTCTTAACTTGTG 23

spu-miR-2004      TCACACACAACCACAGGAAGTT 22
pmi-miR-2004      TCACACACAACCACAGGAAGTT 22
                  **********************

spu-miR-2005      AGTCCAATAGGGAGGGCATTGCA 23

spu-miR-2006      GAGCACACTTGGTAGCGGTGCC 22
pmi-miR-2006      GAGCACACTTGGTAGCGGTGCC 22
                  **********************

spu-miR-2007      TATTTCAGGCAG-TATACTGGTAA  23
pmi-miR-2007      TATTTCAGGCGG-TATACTGGTAA  23
sko-miR-2007      TATTTCAGGCGTTTATACTGGTGA  24
                  **********   ********* *

spu-miR-2008      ATCAGCCTCGCTGTCAATACGA 22
sko-miR-2008      ATCAGCCTCGCTGTCAATACGG 22
                  *********************

spu-miR-2009      TGAGTTGTCCCACAAAGAACAC 22
pmi-miR-2009      TGAGTTGTCCCACAAAGAACAC 22
                  **********************

spu-miR-2010      TTACTGTTGATGTCAGCCCCTT 22
pmi-miR-2010      TTACTGTTGATGTCAGCCCCTC 22
                  *********************

spu-miR-2011      ACCAAGGTGTGCTAGTGATGAC 22
pmi-miR-2011      ACCAAGGTGTGTTAGTGATGAC 22
sko-miR-2011      ACCAAGGTGTGTTAGTGATGAC 22
                  *********** **********

spu-miR-2012      TAGTACTGGCATATGGACATTG 22
pmi-miR-2012      TAGTACTGGCATATGGACATT- 21
sko-miR-2012      TAGTACTGGCATATGGACATTG 22
                  *********************
```

```
spu-miR-2013      TGCAGCATGATGTAGTGGTGTA 21
pmi-miR-2013      TGCAGCATGATGTAGTGGTG-A 22
sko-miR-2013      TGCAGCATGATGTAGTGGTGTG 22
                  ********************

spu-miR-210       TTGTGCGTGCGACAGCGACTGA 22
sko-miR-210       TTGTGCGTGCGACAGCGACTTC 22
                  ********************

spu-miR-219       TGATTGTCCGAACGCAATTCTTG 23

spu-miR-22        TCAGCTGCCCGGTGAAGTGTATA 23
pmi-miR-22        TCAGCTGCCCGGTGAAGTGTAG- 22
                  *********************

spu-miR-242       TTGCGTAGGCGTTGTGCACAGT- 22
pmi-miR-242       TTGCGTAGGCGTTGTGCACAGT- 22
sko-miR-242       -TGCGTAGGCGTTGTGCACAGTG 22
                   *********************

spu-miR-252a      CTAAGTACTAGTGCCGTAGGTT- 22
pmi-miR-252a      CTAAGTACTAGTGCCGCAGGTTG 23
sko-miR-252a      CTAAGTACTAGTGCCGCAGGAGT 23
                  **************** ***

spu-miR-252b      CTAAGTAGTAGTGCCGCAGGTA- 22
pmi-miR-252b      CTAAGTAGTAGTGCCGCAGGTA- 23
sko-miR-252b      CTAAGTAGTAGTGCCGCAGGTAA 23
                  **********************

spu-miR-278       TCGGTGGGACTTTCGTTCGATT 22
pmi-miR-278       TCGGTGGGACTTTCGTTCGATT 22
sko-miR-278       TCGGTGGGACTTTCGTTCGTTT 22
                  ******************* **

spu-miR-29        AAGCACCAGTTGAAATCAGAGC 22
pmi-miR-29        AAGCACCAGTTGAAATCAGAGC 22
sko-miR-29b       TAGCACCATTTGAAATCAGTGT 22
                   ******* ********** *

spu-miR-29b       TAGCACCATGAGAAAGCAGTAT 22
sko-miR-29        TAGCACCATATGAAATCAGTTT 22
sko-miR-29b       TAGCACCATTTGAAATCAGTGT 22
                  *********  ********* *

spu-miR-31a       AGGCAAGATGTTGGCATAGCTG 22
pmi-miR-31a       AGGCAAGATGTTGGCATAGCTG 22
sko-miR-31a       AGGCAAGATGTTGGCATAGCTG 22
                  **********************

spu-miR-31b       AGGCAAGATGCTGGCATAGCT 21
pmi-miR-31b       AGGCAAGATGCTGGCATAGCT 21
                  *********************

spu-miR-33        GTGCATTGTCGTTGCATTGCAT 22
pmi-miR-33        GTGCATTGTAGTTGCATTGCAT 22
                  ********* ************
```

```
spu-miR-34        CGGCAGTGTAGTTAGCTGGTTG 22
pmi-miR-34        TGGCAGTGTGGTTAGCTGGTTG 22
sko-miR-34        TGGCAGTGTGGTTAGCTGGTTG 22
                   ******** ************

spu-miR-375       -TTGTTCGTTCGGCTCGCGTCAA 22
sko-miR-375       TTTGTTCGTTCGGCTCGCGCGA- 22
                   ******************  *

pmi-miR-4171      TGACTCTCTTAAGGTAGCC 19

spu-miR-4847      TAATGATGGCGCGGTGCGGTGC 22

spu-miR-4848a     TGGGTTGAGGCTTTTGGGCAGGA 23

spu-miR-4848b     TGGGTTGAGGCTTTGGGGCAGGA 23

spu-miR-4849      TAATGATGGCGCGGTGCGGTGC 22

spu-miR-4850      TTATCATGACTGTAAACAGGAGG 23

spu-miR-4851      TGATTACTTGCTTTGGAGTTCTT 23

spu-miR-4852      AATTCTATCATTTTGGCTGCAT 22

spu-miR-4853      TAGCTCCGTTGTTGCGTCTTGGTA 24

spu-miR-4854      TGTTGCAGTGACGACTTCGCGC 22

spu-miR-4855      TGTGTAACATCTCATTCAGTGGGT 24

spu-miR-7         TGGAAGACTAGTGATTTTGTTGT 23
pmi-miR-7         TGGAAGACTAGTGATTTTGTTGT 23
sko-miR-7         TGGAAGACTAGTGATTTTGTTGT 23
                  ***********************

spu-miR-71        TGAAAGACATGGGTAGTGAGATT 23
pmi-miR-71        TGAAAGACATGGGTAGTGAGAT- 22
sko-miR-71        TGAAAGACACAGGTAGTGAGAT- 22
                  *********  ***********

spu-miR-79        ATAAAGCTAGGTTACCAAAGATA 23
sko-miR-79        ATAAAGCTAGGTTACCAAAGACA 23
                  ********************* *

spu-miR-9         TCTTTGGTTATCTAGCTGTATG- 22
pmi-miR-9         TCTTTGGTTATCTAGCTGTATGA 23
sko-miR-9         TCTTTGGTTATCTAGCTGTAT-- 21
                  *********************

spu-miR-92a       TATTGCACTTGTCCCGGCCTAC 22
pmi-miR-92a       TATTGCACTTGTCCCGGCCAGC 22
sko-miR-92a       TATTGCACTTGTCCCGGCCTAA 22
                  *******************
```

```
spu-miR-92b       TATTGCACTTGTCCCGGCCTGC 22
pmi-miR-92b       TATTGCACTTGTCTCGGCCAGC 22
sko-miR-92b       TATTGCACTTGTCCCGGCCTGC 22
                  ****************** **

spu-miR-92c       TATTGCACTCGTCCCGGCCTGC 22
pmi-miR-92c       TATTGCACTCGTCCCGGCCTGC 22
sko-miR-92c       TATTGCACTCGTCCCGGCCTGT 22
                  ********************

pmi-miR-92d       TATTGCACTCGTCCCGGCCTAG 22

spu-miR-96        TTTGGCACTAGCACATTTTTGC 21
pmi-miR-96        TTTGGCACTAGCACATTTTTGC 21
sko-miR-96        TTTGGCACTAGCACATTTTTGC 21
                  ********************

spu-miR-981       TTCGTTGTCAACGAAACCTGC 21
```
